# Thermodynamic Irreversibility Analysis of Dual-Skin Chest-Freezer

**DOI:** 10.3390/e24040453

**Published:** 2022-03-25

**Authors:** Vinicius Akyo Matsuda, Álvaro Roberto Gardenghi, Cristiano Bigonha Tibiriçá, Luben Cabezas-Gómez

**Affiliations:** Heat Transfer Research Group, Mechanical Engineering Department, São Carlos School of Engineering, University of São Paulo, Trabalhador São-Carlense Avenue, São Carlos, São Paulo 13566-590, Brazil

**Keywords:** vapor compression refrigeration cycle, transient numerical simulation, thermodynamic irreversibilities, COP, second law efficiency, R290

## Abstract

In this work, a transient analysis of a dual-skin chest-freezer refrigeration system, operating with R290, is studied numerically with the purpose of performing the characterization of the system through the second law of thermodynamics. A mathematical model which accounts for refrigerant mass distribution inside the system is used. In addition, this work addresses the calculation of entropy generation and exergy destruction for characterizing the system performance during its operations. In order to validate the model, a comparison with measured experimental data is performed for both *pull-down* and *on-off* operations. The characterization of the system through the second law of thermodynamics is conducted using two different methods. One consists of a direct calculation of the entropy generation rate and the second one in the calculation of exergy destruction rate. The equivalence of these two methods is used as an indicative of the “correctness” of the performed calculations. The model results agree near 97% with the experimental data used in the comparisons. Entropy generation and exergy destruction results along time for the whole system and in its individual components are characterized with the second law. These results are very useful for improving refrigeration system design.

## 1. Introduction

According to the International Institute of Refrigeration (IIR), the total number of refrigeration, air conditioning and heat-pump systems operating worldwide is estimated at roughly 5 billion units, with 2 billion units being for domestic refrigeration (refrigerators and freezers). In terms of energetic consumption, the refrigeration sector represents roughly 20% of the global energetic consumption [1], including industrial and domestic use. In Brazil, a tropical country, these systems were responsible for about 26.44% of the domestic electric consumption in the year of 2019 (25.72% being for refrigerator alone and 26% 0.72% for freezers) [2]. This fact alone shows that the energetic optimization of this system is an engineering requirement.

One way to achieve such optimization is through the second law of thermodynamics, which may include the analysis of the relation of the system COP and the maximum COP achievable for the system (second law efficiency), and the determination of the thermodynamics irreversibilities that occurs during the system normal operation regimes. For this objective, a robust model that can simulate the system transient and stead-steady regimes and calculate the entropy and exergy balance to characterize the system behavior is necessary.

In this article, a refrigeration system of a dual-skin chest-freezer using propane (R290) as refrigerant is studied numerically, addressing the system’s transient characteristics and performance. The main aims of the study are the development of a numerical model capable of performing the second law analysis of a vapor compression refrigeration cycle along its operation regime.

Skin heat exchangers are increasingly taking part in the market scenario. Such devices are composed of a tube coil heat exchanger passing the internal side of the cabinet walls through an insulation material and fixed to the surfaces. The condenser is fixed on the external surface and the evaporator in the internal one [3]. Ref. [4] affirms that the advantages offered by these heat exchangers include low cost and better utilization of the external space of the refrigerator. However, part of the heat rejection in the condenser is an additional thermal load to the refrigerator, since it is transferred to the inner space of the cabinet through insulation. According to these authors, most domestic freezers are equipped with skin evaporators and a third of them are fitted with a skin condenser.

Propane (R290) is a natural hydrocarbon refrigerant with environmental advantages, such as zero ODP (ozone depletion potential) and very low (less than 10) GWP (global warming potential), and also has good thermodynamic performance [5,6,7,8]. Nowadays, domestic and commercial refrigeration systems with R290 are applied with a renewed interest in small-scale applications due to the aforementioned advantages [9].

**Figure 1:**
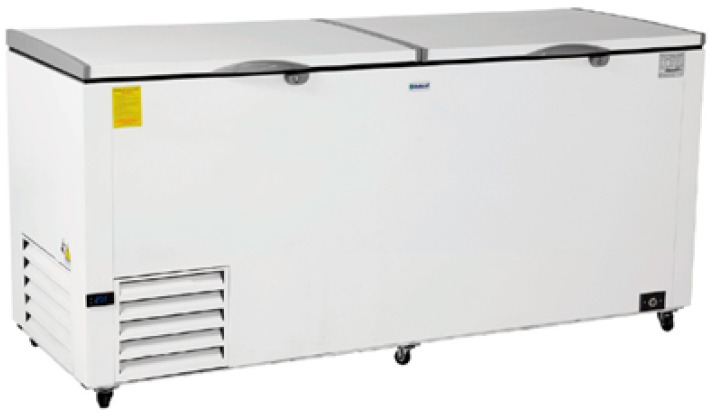
Single compartment dual-skin chest-freezer simulated in this work.

The studied appliance is a single horizontal compartment dual-skin chest-freezer operating with R290, shown in Figure 1. The refrigeration system is composed of a reciprocating compressor, a skin condenser, a capillary tube with no internal heat exchanger, a skin evaporator, 103 g of R290, and electric defrost resistance. The control is assigned to an electrical on/off thermostat with 22.3 to 19.5 °C limits. All the geometric dimensions and other data are presented in [9].

According to the literature review, previous models that address second law aspects of a system operation were developed for lumped parameter analysis [10], much alike the one presented in this work or the lumped parameter moving boundaries model [11], but focusing mainly on the pull-down operating regime. All models encountered in the literature review uses the integrative control volume approach for the system, including the one developed in this work, which can be justified by the fact that vapor compression cycles (VCS) are complex systems. In the present work, the lumped control volume approach published in [9,12,13] is extended by the proposition of very detailed procedures for calculating the temporal entropy generation and exergy destruction rates of the refrigeration system and its components.

The paper is organized as follows. A description of the refrigeration system modeling, including the procedure used for the compressor’s characterization is provided in Section 2. An introduction to the concepts of the second law used in this work, the derivation of the transient exergy destruction rates and entropy generation rates a in a VCS are presented in Section 3, followed by the explanation of the computational algorithm in Section 4. Simulation results using the proposed modified model, including comparisons with experimental data and the previous model of [9] and results regarding the second law of thermodynamics are presented in Section 5. Finally, the conclusions of the work are presented in Section 6.

## 2. Mathematical Model

The mathematical model employed in the simulations performed in this work is the capacitive model developed in [9,12,13]. In summary, this model is composed of sub-models for each main system component (compressor, condenser, capillary tube, evaporator, and cabinet compartment). Each sub-model is a set of ordinary differential algebraic equations (DAEs) that are solved numerically to perform the simulation of the system along its operational time. These equations consists, mainly, on mass and energy conservation laws and heat transfer rates that allow the computation of: component temperatures, fluid evaporation and condensation pressures and their corresponding temperatures, refrigerant mass distribution in the main components, and the degrees of sub-cooling and super-heating. The system performance parameters are also computed, including the system coefficient of performance (*COP*), cooling capacity, consumed compressor electric power, mean consumed energy, and others, for example, see [13].

**Figure 2:**
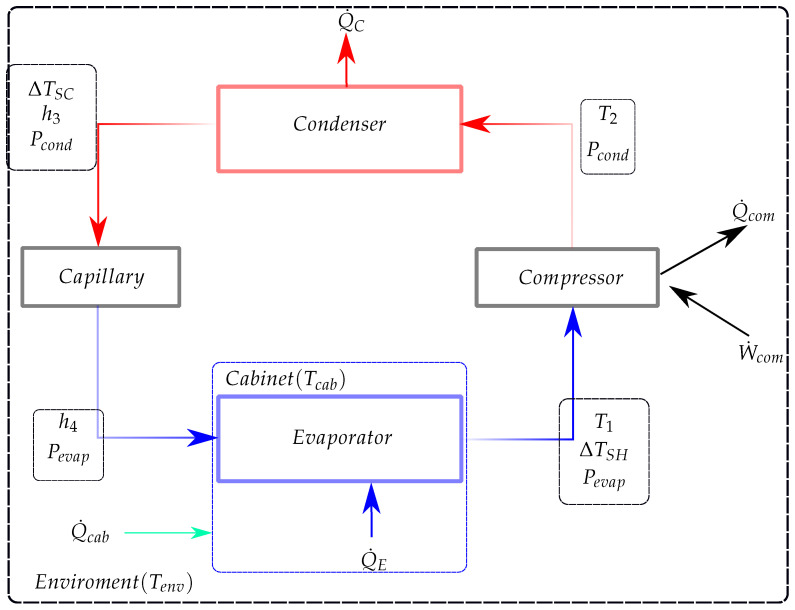
Control volumes and the interaction between the components.

This previous model was properly modified for performing the calculation of component and system transient entropy generation and exergy destruction rates (thermodynamic irreversibilities), as well as the second law efficiency. The modifications performed in the present model in relation to the previous one from [9,12,13] consist of: (i) the calculation of the suction mass flow rate when the compressor is turned off. This mass flow rate is now used in compressor, condenser, and evaporator sub-models; (ii) the calculation of the flashing heat transfer rate in the condenser when the compressor is turned off; (iii) the transient calculations of the entropy generation and exergy destruction rates and of the transient entropy variations. The control volumes adopted for each system component and the interaction between the components are shown in Figure 2. The entropy generation and thermodynamic irreversibilities terms are calculated buy the application of entropy and exergy balances on the same control volumes. The adopted procedure for implementing these equations is presented in Section 3. The original capacitive model from [9,12,13] is denominated as “model A”. The proposed modified model is called “model B”. All models were developed by the same research group.

To enable the implementation of the present modified model the same simplifying hypothesis originally adopted for model A are considered. These are: (i) control volumes of the system components have only one inlet and one outlet; (ii) kinetic and potential energies within and at the open boundaries are neglected; (iii) the thermodynamic and transport properties are uniform in each control volume; (iv) force fields are neglected; (v) delays in transport, pressure losses, and accumulation of refrigerant in the connecting tubes; pressure losses in the condenser and evaporator; spatial temperature variations on the surfaces of the condenser, evaporator, and compressor and within the cabinet compartments are all neglected. The simulations do not consider the opening of doors, following the conditions of the experimental tests. Air infiltration is not taken into account.

**Compressor**: In this work, a variable-speed single cylinder hermetic reciprocating compressor is modeled. The inputs for this model are the inlet temperature and pressure given by $T_{1}$ and $P_{1}$, the inlet specific enthalpy, $h_{1}$, and finally, the outlet pressure, given by $P_{2}$. It is important to note that the inlet and outlet pressures are equal to the evaporation pressure, $P_{evap}$ and the condensation pressure, $P_{cond}$, respectively. In addition, the following input parameters are also necessary: compressor thermal conductance, $UA_{com}$; compressor thermal capacity, $C_{com}$; compressor volumetric efficiency, $\eta_{Vol}$; compressor global (isentropic) efficiency, $\eta_{gl}$; compressor displaced volume, V˙disp, and the compression polytropic exponent, $n_{p}$. The second parameter was obtained experimentally as described in [9,12], as well as the value of $n_{p}$. The compressor efficiencies are obtained by applying the procedures proposed in [14,15]. Finally, there are two other input variables provided at the beginning of the simulations: the ambient temperature, $T_{env}$, and the compressor speed, $N_{RPM}$. From all these inputs, the following outputs are obtained from the compressor model: the compressor mass flow rate, ${\dot{m}}_{com}$; the compressor heat transfer rate through its housing, ${\dot{Q}}_{com}$; the compressor wall temperature, $T_{com}$, and the fluid outlet temperature and enthalpy, $T_{2}$ and $h_{2}$, respectively.

The model is composed by the equations presented Table 1 and operates as follows. First the compressor mass flow rate, ${\dot{m}}_{com}$, is calculated by Equation (1), since this equation depends only on known parameters, including $\eta_{Vol}$. Then, using $\eta_{gl}$, the compressor electric power consumption, ${\dot{W}}_{com}$, is estimated by Equation (2). Note that the global efficiency is computed using an isentropic compression process as reference. For this purpose, the isentropic compressor outlet enthalpy, $h_{2s}$, is determined from the knowledge of $P_{2}$ and inlet fluid specific entropy, $s_{1}$. From the knowledge of the previous compressor state, including the compressor heat transfer rate, ${\dot{Q}}_{com}$, a new compressor housing temperature value is determined by performing a simple energy balance according to Equation (4). Last, the new compressor outlet state is evaluated approximating the compressor fluid outlet temperature by Equation (5), following [9]. The knowledge of $T_{2}$ and $P_{2}$ allows the determination of the compressor outlet state.

It should be noted that in Equation (4), the mass flow rate at the compressor inlet (suction) is denoted as ${\dot{m}}_{suc}$. This variable differs from the system compressor mass flow rate, ${\dot{m}}_{com}$, given by Equation (1). When the system is operating with the compressor turned on, the mass flow rate in the suction side is assumed to be equal to the mass flow rate provided at the compressor discharge (compressor outlet). This is mainly due to the fact that the difference between these two quantities is negligible and only occurs in the very beginning of the process. Nevertheless, when the compressor is turned off, mass may still flow into the compressor inlet and from the compressor outlet. This fact mainly influences the second law transient behavior of the system and the irreversibilities calculation. For the suction side, the method developed by [16] can be used. This procedure consists of the following steps:

1. Calculate the enthalpy of the refrigerant in the shell:
  (6) hshell=m˙suc·h1+VshelldPevapdt+ρshell°·hshell°ΔtVshellρshellΔtVshell
2. Calculate temperature and density of the refrigerant in the shell using the suction pressure and the enthalpy of the refrigerant in the shell.
3. Using the mass conservation law, the mass flow at the compressor suction can be determined as:
  (7) m˙suc=Vshell·ρshell−ρshell°Δt
4. This process must be repeated until a convergence for the suction mass flow rate and the shell enthalpy is reached.

In the above equations, $h_{shell}$ is the specific enthalpy of the refrigerant stored in the compressor suction section (muffler and suction chamber), Vshell is the suction section volume, $\rho_{shell}$ stands for the density of the refrigerant stored in the compressor suction section, and the superscript ${}^{{}^{\circ}}$ represents the assumed initial values or the previous iteration values. $h_{shell}$ differs from $h_{1}$ due to the mixing process that occurs in the compressor shell between the refrigerant already stored in the suction section and the one that is entering into the compressor through the inlet tube. Thus, when the system is turned off, the iterative procedure just explained is employed before the solution of the energy conservation equation, Equation (4), for computing $T_{com}$.

In order to obtain the compressor efficiencies $\eta_{Vol}$ and $\eta_{gl}$ curves for different compressors, the models proposed in [14,15] were employed. In addition, the compressor thermal conductance were approximately determined from the data available in the manufacturer folders. The fitting coefficients were determined through the application of the Levenberg–Marquardt method [17,18] for the minimization of the least square errors. This method was chosen mainly due to the fact that the minimization process is of the unconstrained type. The equations used for the approximation of the efficiency curves and the overall conductance are shown in Table 2. First, the model estimates the efficiencies at a reference fixed speed, indicated by the subscript ref. Then, the efficiencies are corrected to the real compressor speed. The coefficients $a_{n}$, $b_{n}$, ${\dot{W}}_{loss}$, and the thermal conductance $UA_{com}$ are the values to be found. In the process described, $UA_{com}$ is assumed to be independent of the compressor rotation and *k* refers to the isentropic process coefficient.

The consideration of the compressor suction mass flow rate for the compressor’s off period using the iterative procedure developed by [16] and the calculation of the compressor efficiencies and conductance by the methods proposed in [14,15] are two modifications of the model published by [9]. These changes improved the model calculation of the thermodynamic irreversibilities of the system and its components.

**Condenser**: The condenser sub-model is developed for the control volume shown in Figure 2. The input parameters of this model are: the outlet state of the compressor, defined by $h_{2}$ and $T_{2}$; the delivered compressor mass flow rate, ${\dot{m}}_{com}$; the capillary tube mass flow rate, ${\dot{m}}_{cap}$, and the condenser thermal conductance and capacity, $UA_{C}$ and $C_{C}$, experimentally determined in [9]. The model gives the following outputs variables: the condensation pressure and temperature, $P_{cond}$ and $T_{cond}$; the fluid outlet temperature and enthalpy, $T_{3}$ and $h_{3}$; the refrigerant quality, $x_{3}$, (if two-phase); the sub-cooling degree, $\Delta T_{SC}$, the condenser external heat transfer rate, ${\dot{Q}}_{cond}$, the condenser surface temperature, $T_{wc}$, and the refrigerant contain in the condenser, $M_{C}$. The system of equation of the condenser model is displayed in Table 3.

Initially, the heat transfer rate through condenser wall, ${\dot{Q}}_{C}$, is calculated by Equation (13). Then, the new condenser wall temperature is evaluated integrating the energy conservation relation, given by Equation (15), where ${\dot{Q}}_{cond}$ represents the heat transferred from the refrigerant to the condenser wall surface, calculated by Equation (14). In Equation (15) a new term, ${\dot{Q}}_{flash}$, is considered and defined as the flash heat transfer rate, explained later. In the sequence, the mass variation inside the condenser is evaluated using the mass conservation relation, given by Equation (16). The mass contain in the condenser, $M_{C}$, is calculated by the integration of this equation. The calculation of $M_{C}$ needs the knowledge of condenser outlet state that depends on determining the condensation pressure, $P_{cond}$, and the outlet fluid quality, $x_{3}$, and consequently, state.

The outlet fluid quality determination depends on the working regime of the condenser. A condenser analysis leads to the definition of three different conditions based on the mass stored in it. First, with very little refrigerant mass, the condenser is filled completely with super-heated vapor, therefore, at its outlet (state 3), there is only vapor. Due to increase in mass, this regime occurs until the condenser reaches a known mass value, $M_{vc}$, meaning that the outlet state is saturated vapor with quality $x_{3}=1$. A further increase in the refrigerant mass in the condenser raises the pressure, and as a consequence a two-phase region appears in the condenser. In this condition, the outlet is in the two-phase region $0.0\leq x_{3}\leq 1.0$. This regime occurs until a value denoted by $M_{vlc}$, for which the outlet becomes saturated liquid ($x_{3}=0$), is reached. Finally, a further increase in the mass until a value denoted by $M_{lc}$, results, denoting a state where the condenser is filled with sub-cooled liquid, with its inlet being saturated liquid.

These masses are calculated as: MvC=ρvcVC, MvlC=VCαcρvC,sat+(1−αC)ρlC,sat, and MlC=ρlCVC. In these relationships, $\rho_{vC}$ is the average density when $x_{3}=1$ at the condenser outlet, VC is the internal volume of the condenser, $\rho_{vC,sat}$ represents the density of the saturated vapor at the condensation temperature, $\rho_{lC,sat}$ is the density of the saturated liquid in the same condition, $\alpha_{C}$ is the average void fraction when the appearance of sub-cooled liquid is imminent, and $\rho_{lC}$ is the liquid density.

The outlet refrigerant quality is evaluated by Equation (17) considering that this property varies linearly with the refrigerant mass stored in the condenser. If a negative value for the refrigerant quality is achieved through Equation (17), it means a sub-cooled state at the outlet. In this case, the condenser outlet temperature is estimated by performing an energy balance in a differential element in the sub-cooled region. This analysis results in Equation (18) after some algebraic manipulation. This expression depends on the external sub-cooled heat transfer area, $A_{sc}$. This area is calculated based on the external heat transfer area, $A_{C}$, by assuming that the area varies linearly with the mass content, resulting in Equation (19). In this expression, $UA_{sc}$ is the thermal conductance in the sub-cooled region, also calculated through a linearization, see Equation (20). If the outlet state of the condenser is two-phase, this temperature is $T_{3}=T_{cond}$, where $T_{cond}=T_{sat}\left(P_{cond}\right)$.

The refrigerant internal energy can be defined as [19], UC=VC·f1+MC·f2, where $f_{1}=\frac{u_{v,sat}-u_{l,sat}}{v_{v,sat}-v_{l,sat}}$ and $f_{2}=\frac{v_{v,sat}\cdot u_{l,sat}-v_{l,sat}\cdot u_{v,sat}}{v_{v,sat}-v_{l,sat}}$. Since saturated properties only depend on pressure, taking the time derivative of the refrigerant internal energy results in dUCdt=VC·df1dPdPdt+MC·df2dPdPdt+f2·dMCdt. Using the previous expression, and after various algebraic manipulations, Equation (21) is obtained for determining the condensation pressure. The change in the total refrigerant internal energy in the condenser is obtained from a simple energy balance in the condenser control volume, resulting in Equation (22).

**Figure 3:**
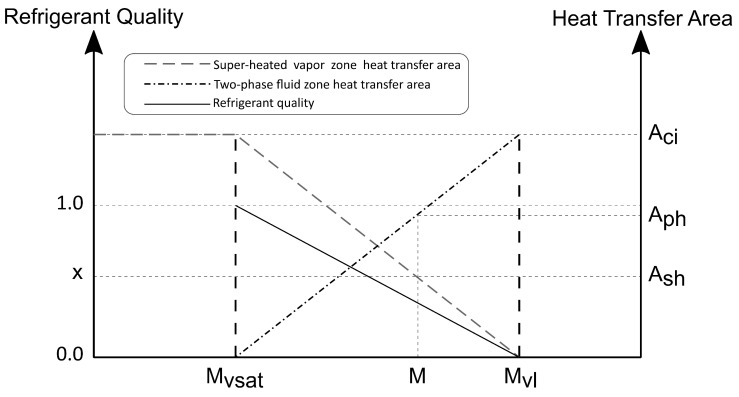
Approximation for the heat transfer areas inside the condenser.

When the compressor is turned off, the mass flow rate through the capillary, ${\dot{m}}_{cap}$, becomes greater than the mass flow rate from compressor discharge, ${\dot{m}}_{com}$, leading to a quick pressure drop on the condenser. It is in this situation that the system experiences a temperature drop due the flash effect. However, due to the complexity of this phenomenon, it is difficult to correctly model it with a lumped model. In order to capture this phenomenon, the heat absorbed by the flashing refrigerant was modeled as an external cold reservoir and is included in the calculation of condenser wall temperature, represented by Equation (15). The heat transfer areas for the super-heated vapor, $A_{sh}$ and the flashing fluid, $A_{flash}$, were calculated by assuming a linear variation of these areas with the mass stored inside the system in super-heated state and the mass that must undergo the flashing process. Starting with little refrigerant mass, the condenser is filled completely with super-heated vapor, therefore, $A_{sh}$ is the total internal area of the condenser ($A_{sh}=A_{ci}$). As the mass content increases, a known mass value, $M_{vsat}$, is reached. This means that the outlet state is saturated vapor with $x_{3}=1.0$. A further increase in the refrigerant mass in the condenser results in the appearance of a two-phase region, in this condition, $A_{sh}$ starts to decrease. This regime occurs until a value denoted by $M_{vl}$, for which the outlet becomes saturated liquid ($x_{3}=0$) and $A_{sh}$ reaches a negligible value ($A_{sh}=0$), as shown in Figure 3.

In the first moments, $T_{cond}$ is still be higher than $T_{wc}$, and it is not necessary to consider the term ${\dot{Q}}_{flash}$. When $T_{cond}$ becomes lower than $T_{wc}$ due to the condenser pressure decrease, it is assumed that the region of the condenser occupied by the refrigerant in the two-phase state undergoes the flashing phenomenon, receiving heat from the condenser wall and cooling the condenser wall, i.e., the condenser component. This exchanged heat rate is denoted by ${\dot{Q}}_{flash}$ and is calculated through Equation (24). It is easy to note that this equation is similar to Equation (14) but employs the flashing heat transfer area, $A_{flash}$, which is calculated by Equation (26). For calculating ${\dot{Q}}_{cond}$ using Equation (14) in the flashing process, the bulk temperature is computed as the average temperature between the inlet an outlet temperatures in the condenser, and the heat transfer area should be calculated as given by Equation (25). It is important to note that when the system is operating with the compressor on, the quantity ${\dot{Q}}_{flash}$ is null, since there is no flash evaporation during this period. The inclusion of this flashing heat transfer rate (see Equation (24)) is the other modification of the previous published model by [9].

The derivatives of $f_{1}$ and $f_{2}$, calculated through the fitting polynomials, and its coefficients are shown in Table 4 and Table 5, respectively.

**Capillary tube**: For the simulated refrigerator, the capillary model is rather simple, since it is considered an adiabatic process wit $h_{4}=h_{3}$. The model inputs are $P_{cond}$ and $P_{evap}$, the specific volume at the capillary tube inlet, $v_{3}$, and the degree of sub-cooling, $\Delta T_{sc}$. Thus, the model is applied for calculating the refrigerant quality at the capillary outlet, $x_{4}$, and the capillary mass flow rate, ${\dot{m}}_{cap}$, using Equation (29) [12,19].

(29) $${\dot{m}}_{cap}=a\cdot \sqrt{\frac{P_{cond}-P_{cap,out}}{v_{3}}}+b\cdot \Delta T_{sc}+c$$

The coefficients *a*, *b*, and *c* were determined through experimental results (see [9,12]) being adjusted as: *a* = 0.0050416, *b* = 0.3460787, and *c* = 0. At the outlet of the capillary tube, a critical flow is possible due to the high acceleration of the fluid in the device. Thus, the Fauske criterion [20,21] was implemented to determine the critical pressure, $P_{crit}$. The effective pressure at the outlet of the tube is calculated as $P_{cap,out}=max\left(P_{evap},P_{crit}\right)$.

**Evaporator**: The evaporator sub-model is developed for the control volume shown in Figure 2. The model inputs are: the capillary tube outlet enthalpy $h_{4}$, the mass flow rates ${\dot{m}}_{com}$ and ${\dot{m}}_{cap}$, and the experimentally determined (see [9]) evaporator thermal conductance and capacity, $UA_{E}$ and $C_{E}$, respectively. The model gives as its outputs the evaporation pressure and temperate, $P_{evap}$ and $T_{evap}$, the evaporator temperature and enthalpy, $T_{5}$ and $h_{5}$, the refrigerant quality (if tow-phase), $x_{5}$, the evaporator surface temperature and heat transfer rate, $T_{we}$ and ${\dot{Q}}_{E}$, the super-heating degree, $\Delta T_{SH}$, and the refrigerant mass stored in the evaporator, $M_{E}$. The equations that compose the evaporator sub-model are shown in Table 6.

Initially, the heat transfer through evaporator wall, ${\dot{Q}}_{E}$, is calculated by Equation (30). Then, the new evaporator wall temperature is evaluated integrating the energy conservation equation, given by Equation (32), where ${\dot{Q}}_{evap}$ is the heat transferred to the refrigerant from the evaporator wall surface, calculated by Equation (31). In the sequence, the mass variation inside the evaporator is evaluated using the mass conservation equation, given by Equation (33). The mass stored in the evaporator, $M_{E}$, can be found integrating this equation. The calculation of $M_{E}$ needs, similarly to the condenser, the knowledge of evaporator outlet state 5. The determination of state 5 depends on the calculation of evaporation pressure, $P_{evap}$, and of the fluid quality, $x_{5}$, at the evaporator outlet.

Three different conditions of the evaporator can be defined based on the mass stored in it. Initially, with the system storing a great amount of refrigerant, the evaporator is completely filed with sub-cooled liquid. Decreasing the refrigerant mass stored in the system until a quantity denoted by $M_{le,sat}$, and given by MlE=ρlEVE, the outlet becomes a saturated liquid, i.e., $x_{5}=0.0$. Then, a continuous diminution of the refrigerant mass stored in the evaporator leads to a pressure decrease, resulting in the presence of a two-phase state ($0.0\leq x_{5}\leq 1.0$) in the evaporator. When the evaporator outlet reaches the condition of saturated vapor, i.e., $x_{5}=1.0$, the refrigerant mass stored in the evaporator is denoted by $M_{lve}$, given by MvE,sat=[αE·ρvE,sat+(1−αE)·ρlE,sat]VE. A further decrease in the refrigerant mass leads to a state were the evaporator is entirely filled of super-heated vapor, for which the mass stored is denoted by $M_{vE}$, being given by MvE=ρvEVE. In these relationships, VE is the total internal volume of evaporator, $\rho_{le}$ is the average density when the quality at the evaporator outlet is 0, $\rho_{vE,sat}$ represents the density of the saturated vapor at the evaporation temperature, $\rho_{lE,sat}$ is the density of the saturated liquid in the same condition, $\alpha_{E}$ is the average void fraction when the appearance of super-heated vapor is imminent, and $\rho_{v,E}$ is the vapor density.

The outlet refrigerant quality is evaluated by Equation (34), considering that this property varies linearly withe refrigerant mass stored in the evaporator. If, according to Equation (34), a value greater than one is achieved, there is super-heated vapor at the outlet. In order to completely determine the outlet thermodynamic state, an energy balance in a differential element in the super-heated region is performed. This analysis results in Equation (35). In the previous expression, $c_{p,v}$ is the specific heat of the vapor and $UA_{sh}$ is the thermal conductance in the super-heated region, calculated through a linearization by Equation (36). The external super-heated heat transfer area, $A_{sh,E}$, is calculated through the external heat transfer area, $A_{E}$, by assuming that the area varies linearly with the mass content, resulting in Equation (37). If $0\leq x_{5}\leq 1$, the outlet state of the evaporator is two-phase, and the temperature is $T_{5}=T_{evap}$, where $T_{evap}=T_{sat}\left(P_{evap}\right)$.

The refrigerant internal energy can be defined as [19], UE=VE·f1+ME·f2 using the previously defined $f_{1}$ and $f_{2}$. Since saturated properties only depend on pressure, taking the time derivative of the refrigerant internal energy results in $\frac{dU_{E}}{dt}=V\cdot \frac{df_{1}}{dP}\frac{dP}{dt}+M_{c}\cdot \frac{df_{2}}{dP}\frac{dP}{dt}+f_{2}\cdot \frac{dM_{E}}{dt}$. Using this expression, and after various algebraic manipulations, Equation (38) is obtained for calculating the evaporation pressure. The derivatives of $f_{1}$ and $f_{2}$ were calculated through a polynomial fitting shown in Table 4, and were also used in the condenser model. The change in the total refrigerant internal energy in the evaporator is obtained from a simple energy balance in the evaporator control volume, resulting in Equation (39). In Equation (39), the term $\dot{m_{com}}$ is changed by $\dot{m_{suc}}$ when the compressor is shut down.

**Cabinet**: The inputs for this model are: the evaporator wall temperature, $T_{we}$, the heat transfer rate to the evaporator wall, ${\dot{Q}}_{E}$, the cabinet conductance and thermal capacity, $UA_{cab}$ and $C_{cab}$, experimentally determined, and the goods’ conductance and thermal capacity, $UA_{g}$ and $C_{g}$, respectively. The variable $UA_{g}$ is calculated using the appropriate heat transfer coefficients, following [9,13]. The outputs of the model are the temperature of the air inside cabinet, $T_{cab}$, and the goods’ temperature, $T_{g}$. The goods’ temperature is calculated when the refrigerator is simulated with thermal loads. The equations that compose the cabinet sub-model are shown in Table 7.

First, the heat transferred to the cabinet from the ambient is computed by Equation (40). Then, applying an energy conservation on the cabinet control volume the cabinet temperature time derivative, given by Equation (41), is obtained. Integrating this equation, $T_{cab}$ is calculated. It is important to be able to consider the presence of thermal loads in the system. This is performed considering the heat transfer rate, ${\dot{Q}}_{g}$, defined by Equation (42) and solving Equation (43) for determining $T_{g}$. Note that the term, ${\dot{Q}}_{g}$, displayed in Equation (41), is considering only when goods are inside the cabinet.

## 3. Second Law Analysis

In this section, the methodology for calculating the entropy generation and thermodynamic irreversibilities (exergy destruction) of the refrigeration system and its components is presented. The total entropy generation rate, ${\dot{S}}_{ger}$, for each component is calculated from Equation (44), which is derived from an entropy balance for a control volume, following [22,23].

(44) $${\dot{S}}_{ger}=\sum {\dot{m}}_{out}s_{out}-\sum {\dot{m}}_{in}s_{in}-\sum \frac{{\dot{Q}}_{k}}{T_{k}}+\frac{dS_{CV}}{dt}\geq 0$$

In Equation (44) the subscripts *k*, in, out, ger, and CV stand for boundary, inlet, outlet, generated, and control volume, respectively. The therm ${\dot{Q}}_{k}$ means heat transfer interaction at a particular *k* boundary of control volume. The rate of exergy destruction or thermodynamic irreversibilities are calculated by Equation (45), obtained from an exergy balance for a control volume (see [22,23]).

(45) X˙des=∑1−TenvTk · Q˙k−W˙−Penv·dVdt+∑m˙inψin−∑m˙outψout−dXCVdt≥0

In Equation (45) the subscript env stands for the environment reference values, *X* represents the total exergy of the control volume, while ψ is the specific exergy of the flowing streams. The destroyed exergy and generated entropy are equivalent quantities that are related through the Gouy–Stodola theorem [23]. Using this relation, it is possible to calculate the thermodynamic irreversibilities of the system through the knowledge of system entropy generation. This relation in terms of rates is expressed as:

(46) $${\dot{X}}_{des}=T_{env}\cdot {\dot{S}}_{ger}$$

In the present work, the thermodynamic irreversilities are calculated from Equations (45) and (46) for validating the proposed procedure. Another core concept derived from the second law of thermodynamics is the concept of second law efficiency, $\eta_{II}$, which relates with system efficiency and the maximum achievable thermodynamic efficiency for the system operating under the same condition. SLE can be expressed by:

(47) $$\eta_{II}=1-\frac{X_{des}}{X_{avail}}$$

The subscripts avail and des mean available and destroyed, respectively. The system efficiency given by Equation (47) can be rewritten using the reversible work performed by the system, which results in:

(48) $$\eta_{II}=\frac{{\dot{W}}_{rev}}{{\dot{W}}_{rev}+{\dot{X}}_{des,sys}}$$

where the subscripts rev and sys stand for a reversible process and the system, respectively, and the reversible work is calculated as:

(49) $${\dot{W}}_{rev}=\left[1-\frac{T_{cab}}{T_{env}}\right]{\dot{Q}}_{E}$$

Equations (47) and (48) can be used to evaluate the second law refrigeration system performance. The second law analysis of the system is consistent with the superposition concept, allowing the overall analysis of the system from an individual analysis for each component. The application of this concept results in the following equation for the system exergy destruction and entropy generation rates, ${\dot{X}}_{des}$ and ${\dot{S}}_{gen}$, calculation:

(50) $${\dot{X}}_{des,sys}={\dot{X}}_{des,com}+{\dot{X}}_{des,C}+{\dot{X}}_{des,cap}+{\dot{X}}_{des,E}$$

(51) $${\dot{S}}_{gen,sys}={\dot{S}}_{gen,com}+{\dot{S}}_{gen,C}+{\dot{S}}_{gen,cap}+{\dot{S}}_{gen,E}$$

Due to the equivalence of these concepts, two different paths can be taken to evaluate these quantities. The exergy destroyed in the system can be evaluated through an exergy balance (Equation (50)) and then converted into entropy generation rate, or, the entropy generated can be evaluated through an entropy balance (Equation (51)) and then converted into exergy destroyed using Equation (46). In the following subsections, the presented general formulation for the thermodynamic irreversibilities calculation is applied for each component.

### 3.1. Compressor

For the compressor, a quasi-steady model is adopted, which neglects all time derivatives with respect to the refrigerant mass flow rate. The equations of compressor entropy generation and exergy destruction rates are presented in Table 8. The compressor control volume uses extended boundaries to the environment resulting in Equation (52) for the rate of destroyed exergy. The entropy generated in the compressor is calculated using the same control volume and quasi-steady hypothesis, leading to Equation (53).

In both equations, Equations (52) and (53), the subscript *hous* refers to the “solid” part of the system, which can not be treated in a quasi-steady state. The term $\frac{dX_{com,hous}}{dt}$ is the total exergy change with time of the control volume, and can be calculated by (Equation (54)), where the term relative to change in the system entropy over time can be calculated by its definition, leading to Equation (55).

It is also important to note that when the system is operating with the compressor on, the mass flow rate in the suction and discharge lines is the same and equal to ${\dot{m}}_{com}$. This assumption can be used since values of ${\dot{m}}_{com}$ evolve to the steady-state regime much faster than the system as a whole.

Finally, the compressor housing total internal energy variation can be calculated through the energy conservation relation, given by Equation (4).

### 3.2. Capillary Tube

A similar quasi-steady hypothesis assumed for the compressor can be made for the capillary tube. The equations of capillary tube entropy generation and exergy destruction rates are presented in Table 9. Since the process that the capillary tube undergoes is assumed to be isenthalpic, the exergy destruction rate for this component is reduced to Equation (56), and the entropy generation rate is given by Equation (57).

### 3.3. Heat Exchangers

The control volume boundaries of these components were expanded to the environment, as was assumed for the compressor model. By doing this, the heat transfer rate ${\dot{Q}}_{wall}$ is relative to the heat exchanged with the environment at $T_{env}$, and the time derivatives relative to the heat exchangers walls is considered in the governing equations. The equations for heat exchangers entropy generation and exergy destruction rates are presented in Table 10.

The exergy balance for both heat exchangers results in Equation (58) and the entropy balance results in Equation (59). Its important to note that in both equations the inlets and outlets are those shown in Figure 2. Furthermore, the time derivatives, $\frac{d}{dt}X_{VC}$ and $\frac{d}{dt}S_{VC}$ include the refrigerant and solid components of the control volume. Considering the fact that internal energy and entropy are additive properties, $\frac{d}{dt}X_{VC}$ can be calculated from its definition by Equation (60). Note that in this relation the subscripts ref and wall are employed for denoting the refrigerant and the solid components, respectively. For the solids components, a calculation similar to that performed for the compressor can be employed. The change in the solids wall internal energy can be found by simply multiplying Equations (15) and (32) by their respective thermal capacitances for the condenser and evaporator, respectively. The solids’ total entropy change with time is given by Equation (61). The time variation of internal energy for the refrigerant side is given by Equations (22) and (39) for the condenser and evaporator, respectively.

The refrigerant entropy change, $\frac{dS_{ref}}{dt}$, can be written as shown in Equation (62). Applying the chain rule results in Equation (63), where two main contributions can be seen. The first term of Equation (63) refers to the change in the entropy due to the variation in the mass stored in the system and can be estimated through Equation (16) for the condenser and Equation (33) for the evaporator. The second term is relative to the variation in the specific entropy of the mass of refrigerant stored in the heat exchanger, $\frac{ds}{dt}$.

(62) $$\frac{dS_{ref}}{dt}=\frac{d}{dt}\left[M\cdot s\right]$$

(63) $$\frac{dS}{dt}=s\cdot \frac{dM}{dt}+M\cdot \frac{ds}{dt}$$

The estimation of $\frac{ds}{dt}$ is difficult and essential in this work. Two main approaches can be used in order to calculate this term. The first one is a quasi-steady or path-independent method, shown in Equation (64). Basically, this method uses a finite difference method to estimate the entropy derivative. The disadvantages of this method are related to the small time step, Δt, needed in order to achieve precise results. The second approach is the one developed in [24], and is given by Equation (65). This method assumes that the entropy is a continuous and differential function, and that it is a function of two thermodynamics properties, $var_{1}$ and $var_{2}$. Taking into account what was discussed in [24], the second approach was selected. Choosing as the state variables the saturation pressure, $P_{sat}$, and the bulk specific enthalpy, *h*, Equation (65) can be rewritten into Equation (66).

(64) $$\frac{ds}{dt}=\frac{s_{i}-s_{i-1}}{\Delta t}$$

(65) $$\frac{ds}{dt}={\left(\frac{\partial s}{\partial var_{1}}\right)}_{var_{2}}\cdot \frac{dvar_{1}}{dt}+{\left(\frac{\partial s}{\partial var_{2}}\right)}_{var_{1}}\cdot \frac{dvar_{2}}{dt}$$

(66) $$\frac{ds}{dt}={\left(\frac{\partial s}{\partial h}\right)}_{P_{sat}}\cdot \frac{dh}{dt}+{\left(\frac{\partial s}{\partial P_{sat}}\right)}_{h}\cdot \frac{dP_{sat}}{dt}$$

The partial derivatives, ${\left(\frac{\partial s}{\partial h}\right)}_{P_{sat}}$ and ${\left(\frac{\partial s}{\partial P_{sat}}\right)}_{h}$, presented in Equation (66), can be estimated using the CoolProp library, ref. [25]. The saturation pressure derivative, $\frac{dP_{sat}}{dt}$, is known through Equations (21) and (38) for the condenser and the evaporator, respectively. The only unknown term in Equation (66) is the enthalpy derivative, $\frac{dh}{dt}$. This quantity can be calculated using the enthalpy definition given by Equation (67). Taking the time derivative of this expression results in Equation (68), from which the enthalpy derivative can be found.

(67) dh=du+Pdv+vdP

(68) $$\frac{dh}{dt}=\frac{du}{dt}+P_{sat}\cdot \frac{dv}{dt}+vs.\cdot \frac{dP_{sat}}{dt}$$

In order to use Equation (68), three other derivatives must be calculated. The first one is the specific internal energy derivative, $\frac{du}{dt}$. It can be calculated through the refrigerant internal energy equations: Equations (22) and (39). By knowing that U=M·u, Equation (69) can be written. Isolating $\frac{du_{c}}{dt}$ results in Equation (70).

(69) $$\frac{dU}{dt}=\frac{d}{dt}\left(M\cdot u\right)=u\cdot \frac{dM}{dt}+M\cdot \frac{du}{dt}$$

(70) $$\frac{du}{dt}=\frac{1}{M}\cdot \left[\frac{dU}{dt}-u\cdot \frac{dM}{dt}\right]$$

In the sequence, the specific volume derivative, $\frac{dv}{dt}$, must be estimated. This can be performed through the mass derivative $\frac{dM}{dt}$. Knowing that the mass is given by M=V·ρ, the mass change in the system can thus be written as in Equation (71). Taking the time derivative of the expression shown above and isolating the density derivative results in Equation (72). Now, substituting ρ=1/v into this expression and rearranging it, the specific volume derivative is found (see Equation (73)).

(71) dMdt=V·ddt[ρ]

(72) dρdt=1V·dMdt

(73) dvdt=dMdt·1V·v2

Finally, by substituting Equations (70) and (73), and the pressure derivative, Equation (21) or (38) into Equation (68), the specific enthalpy derivative is found. Substituting this expression into Equation (66), the specific entropy derivative is calculated.

## 4. Computational Algorithm

**Figure 4:**
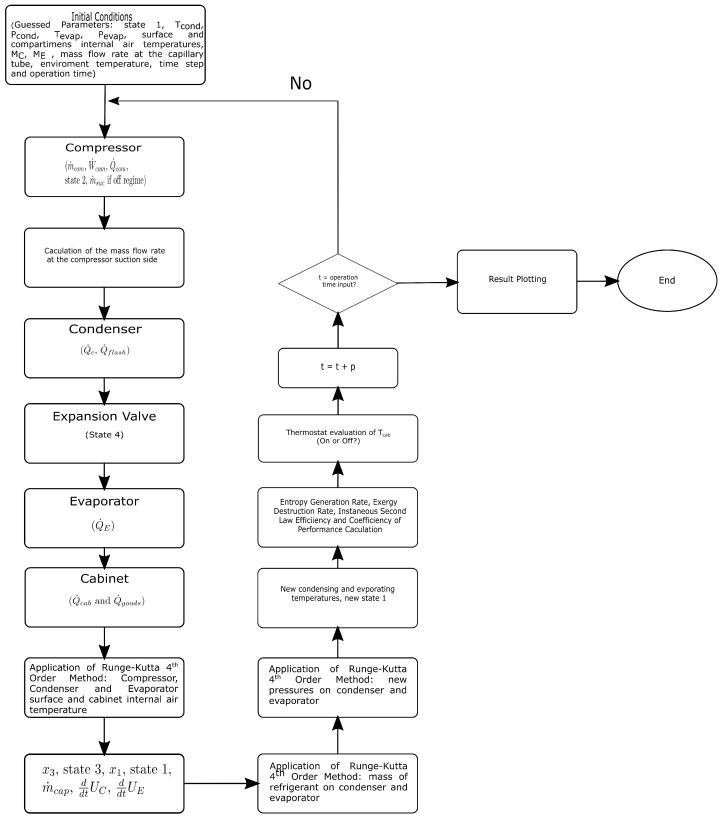
Numerical Algorithm.

The computational algorithm is shown in Figure 4. To start the simulations, the user needs to provide some input parameters, such as: the environment temperature, the compressor speed, the type of control used (on-off or other type as the proportional), the operating time, the desired time step, and the presence or absence of a thermal load in the system. The thermal capacitances and conductances are also input parameters settled by the user. Before the beginning of the calculations, the values of various variables are settled as guesses ($P_{cond},P_{evap},T_{cond},T_{evap},M_{E},M_{C}$, component surface, and internal air temperatures) (see Figure 4). The calculation starts with the compressor sub-model. Thus, the mass flow rate imposed by the compressor, the electrical power input or compressor consumed power, the heat rejected by the compressor housing, and the state 2 are determined. In sequence, the mass flow rate at the compressor inlet is estimated using the iterative model explained in Section 2. Then, the heat transfer rate in the condenser is determined, followed by the capillary tube outlet state 4, then the heat transfer rate in the evaporator is estimated, and finally the cabinet sub-model heat transfer rate and the thermal load heat transfer (if considered) are calculated.

Then, the 4th order Runge–Kutta method is applied to solve the algebraic differential equation system (DAEs) and estimate the components’ surface temperatures and cabinet indoor air temperature. After that, a new mass flow rate of the capillary tube, the state 3 and state 1, are determined. Then, the DAEs for the refrigerant mass inside the condenser and evaporator are solved, and in sequence the new condensation and evaporation pressures are calculated, also using the 4th order Runge–Kutta method. Once all these calculations are performed, the second law parameters calculation is realized, computing the rates of entropy generation, exergy destruction, and instantaneous COP and $\eta_{II}$ in the current time step. Finally, the cabinet temperature verification is performed, in order to known if its necessary to shut down the compressor for the on-off control.

## 5. Results

In this section, the obtained simulation results with Model B are provided. First, in Section 5.1, simulation results for the *Pull-Down* freezer operation are provided. The results are compared with experimental data and with the simulation results obtained with the previous model A (see [9,12]) in order to validate the proposed modeling modifications. Then, in Section 5.2, the results obtained for the cyclic *On–Off* freezer operation are presented. These results are also compared with the experimental and numerical results from model B [9,12] for a second validation of the proposed changes in the simulation methodology. In both sections, results regarding the proposed second law analysis are presented. This includes the presentation of simulated values for the rates of entropy generation and exergy destruction of the system and its main components. Besides this, the entropy generated within each heat exchanger is shown in detail. Last, in Section 5.3, an analysis of the freezer performance when thermal loads are put inside it, considering three different values of ambient temperature ($T_{env}=25,32$ and 43 °C), is presented.

The main inputs parameters considered in the simulations can be consulted in [9]. In Table 11 the employed input values of the thermal capacitances and conductances for the system components are presented. All these data were experimentally obtained by [9,12], excepting the compressor thermal conductance that was calculated by the method described in Section 2. The simulated temperature values are shown in °C units. However, in the calculation of relative and RMS errors and in the second law relations K units are used.

### 5.1. Pull-Down

The pull-down simulations were performed for $T_{env}=25$ °C, 4500 rpm and a refrigerant charge of 103 g of R290.

**Figure 5:**
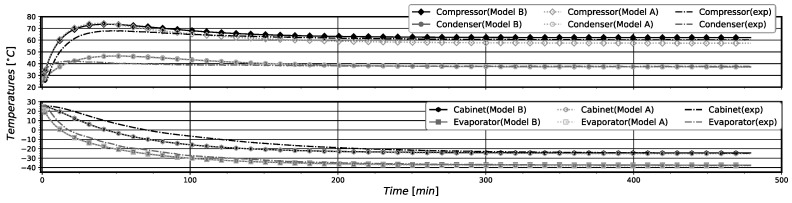
Pull-down components temperatures comparison with experimental data.

**Figure 6:**
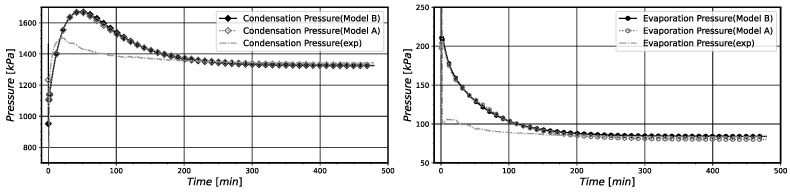
*Pull-Down* saturation pressures comparison with experimental data.

In general, the pull-down simulations with models A and B showed a good agreement with experimental data, correctly estimating the steady-state regime for the given operating conditions, as can be seen in Figure 5 and Figure 6. It is important to note that when the compressor is just turned on, the simulations present a higher divergence from the experimental data. The higher differences were obtained for the simulated pressure values, displayed in Figure 6. This behavior is mostly due to the difficulties in correctly estimating the mass distribution in the system during the first minutes of system operation. In fact, after this initial period of operation the simulation results almost coincide with the experimental data. In Table 12, the relative errors at the steady-state regime and the root mean square (RMS) errors for the entire time series for the component temperatures and saturation pressures in relation to the experimental data are presented. The stationary regime was attained when the system reached 479 min of operation. The calculated relative errors for the two models are low. Both models presented RMS errors bellow 2% for all component temperatures, but presented higher RMS for the evaporation pressure (about 22%). It is possible to notice that model B presents a slightly better agreement with the experimental data regarding the compressor, evaporator, and cabinet temperatures, while showing slightly worst results for the condenser.

The relatively higher RMS errors for the evaporation pressure occur mainly due to the differences in the evaporation pressure for the first 100 min of operation. In this time interval, the evaporation experimental pressure quickly drops and the simulated ones decay more slowly, leading consequently to high RMS errors. The correct simulation of the pull-down operation in the first minutes of operation is complex due to the difficulties in simulating the real transient pressure variations of the system. The main reason that seems to provoke the obtained high RMS errors for the evaporation pressure is the difficulty for correctly estimating the initial refrigerant mass distribution inside the system components (mainly in the heat exchangers) of the real system. This difficulty influences the model capability for providing reliable simulation results of the saturation pressures for the pull-down operation in the first time intervals. This especially occurs for the evaporator, which is the component with higher refrigerant content at the beginning of the pull-down operation. However, as can be noted, these errors do not affect the system performance parameters’ numerical estimation nor the simulated component temperatures in relation to the experimental measured values. See the results displayed in Figure 5 and Table 12.

**Figure 7:**
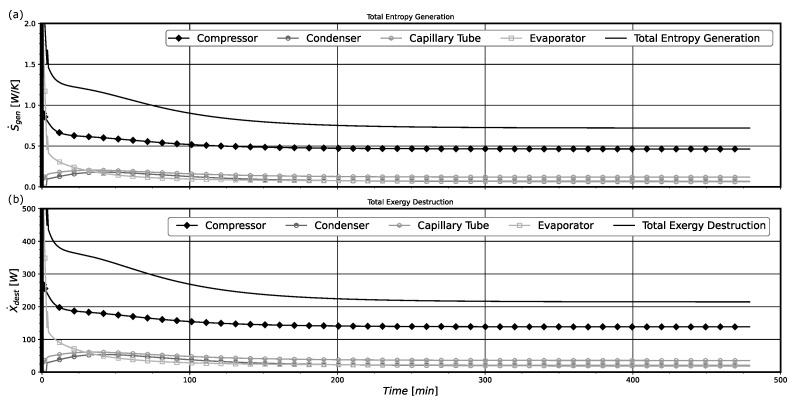
(**a**) Pull-down entropy generation rate of the system and its components. (**b**) Pull-down exergy destruction rate of the system and its components.

The rates of entropy generation and exergy destruction in the system along its operation are presented in Figure 7 for model B. Comparatively, it is possible to notice that both curves present identical behaviors, with the exergy destruction curve being related to the entropy curve through the environmental temperature, accordingly to Equation (46).

The compressor is the principal source of system irreversibilities, followed by the capillary tube and then by the heat exchangers. However, at the very beginning, the evaporator was producing more entropy than the compressor. This effect could be caused by the initial temperature field properly of the pull-down operation. As the temperature differences in this component are very high when the system starts to operate, the thermodynamic irreversibilities results in the highest one. The system presents greater irreversibilities in the first period of operation for almost 50 min. Then, they decay over time, reaching their minimum values as the system goes to the steady-state regime. Actually, after 200 min of operation, the thermodynamic irreversibilities stay constant. The main reason for this behavior is the almost constant values of system pressures, temperatures, and mass flow rates, that are characteristic of the pull-down operation near the steady-state regime conditions.

**Figure 8:**
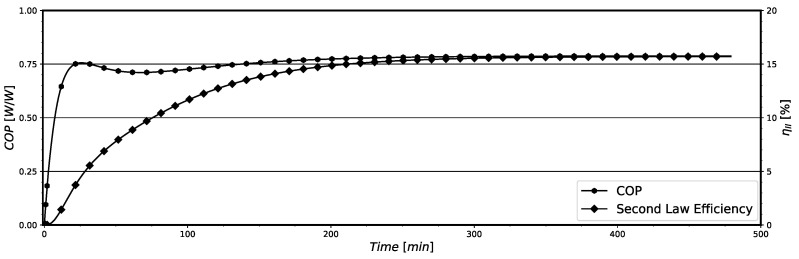
System instantaneous $\eta_{II}$ and COP on pull-down for model B.

**Figure 9:**
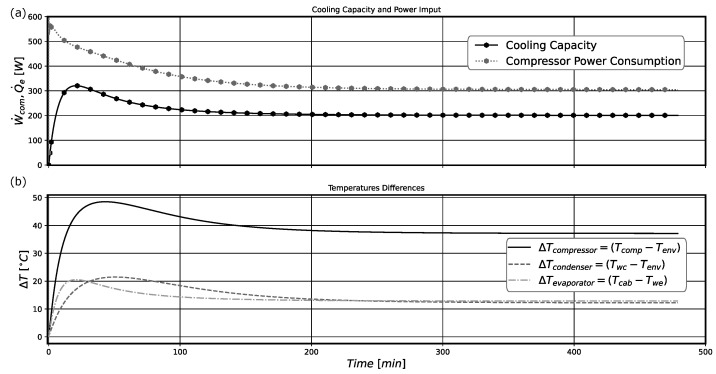
(**a**) Cooling capacity and compressor electrical power consumption. (**b**) Temperature differences. Both results are for model B.

The instantaneous values for the $\eta_{II}$ and COP, estimated along the system operation, are shown in Figure 8 for model B. By comparing Figure 7 and Figure 8, it is noticeable that when the system presents its maximum irreversibilities (in the initial period of operation) it presents its lower values for $\eta_{II}$ and COP. However, both quantities present a notable difference in their behavior in the first 200 min of operation. The system COP only present very low values at the first 20 min of operation, approximately. After this period, the system almost attains the COP value representative of the steady-state pull-down regime. The physical reason for this behavior is that the instantaneous cooling capacity also increases very fast, leading to increasing COP values in a short period of time (see the results presented in Figure 9a). Otherwise, the thermodynamic irreversibilities depend more on the temperature differences and mass flow rate sudden variations in the system components, which are high in this initial period of operation, presented in Figure 9b, and produce higher thermodynamic irreversibilities as previously presented in Figure 7. This behavior leads to a much slower increase in system $\eta_{II}$ in comparison with that for COP. Actually, these high temperature differences between component walls and the refrigerant fluid lead to an abrupt increase in the cooling capacity, improving the system COP.

### 5.2. On–Off

The on-off simulations were performed for $T_{env}=25$ °C, 4500 rpm and 103 g of R290. The temperature limits for the on-off control were set to −19.5 °C and −22.3 °C, as the upper and lower temperatures, respectively.

**Figure 10:**
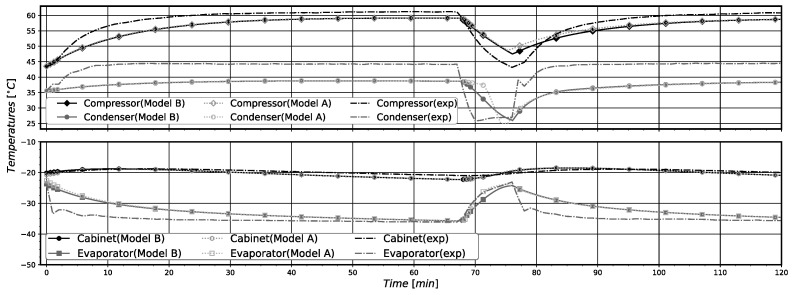
On-off components’ temperatures comparison with experimental data.

**Figure 11:**
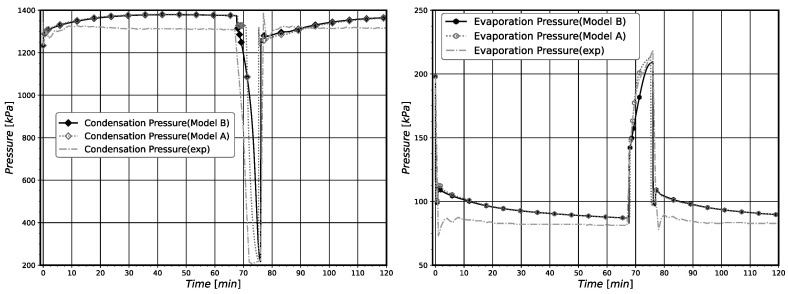
On-off saturation pressures comparison with experimental data.

The results for component temperatures and saturation (condensation and evaporation) pressures achieved with both models, together with the experimental data, are shown in Figure 10 and Figure 11, respectively. In the periods where the compressor is operating (turned on), it is possible to notice that models A and B present similar behavior. The major difference between the models occurs when the compressor is turned off. Solely analyzing $T_{wc}$ variation with time for model A, it can be seen that $T_{wc}$ stays in a higher value for longer periods, and then presents a similar decay as the experimental temperature, reaching 25.0 °C, which is 1.8 °C bellow the experimental temperature value. For the model B, $T_{wc}$ constantly decays with time but does not present the abrupt decay of the experimental temperature. $T_{wc}$ reaches a value of 26.0 °C, resulting in an absolute error of 0.8 °C when the system starts again. In general, a good agreement with experimental data can be seen for temperatures and pressures. Yet, it is important to note that when the compressor is turned off, both the pressure and the temperature decay less abruptly than the experimental values does. This behavior may be a consequence of the slight differences in the mass distribution inside the system.

In Table 13, the averaged performance parameters, estimated along the entire system operation cycle, are presented. Both models show similar results, presenting close values for all performance parameter evaluated. The highest relative errors were found for ${\dot{W}}_{avg}$, being equal to 2.82% for model A and 2.05% for model B. These results validate the proposed model B modifications. In Table 14, the calculated RMS error for the entire time series and the components temperatures are provided. Both models presented similar results. All components’ temperatures presented RMS errors lower than 3%.

**Figure 12:**
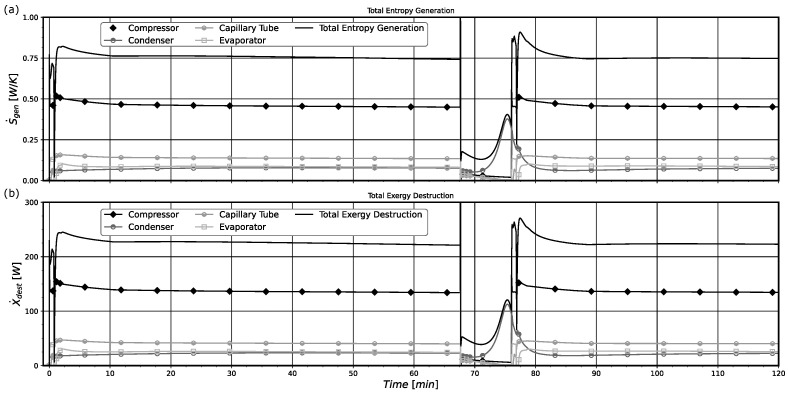
(**a**) On-off total entropy generation rates of the system and its component. (**b**) On-off total exergy destruction rates of the system and its components. Results obtained for model B.

In Figure 12, the rates of entropy generation and exergy destruction for the whole system and its components with time are shown for model B. The compressor component again is the main source of thermodynamic system irreversibilities during the on period, followed by the capillary tube and the heat exchanges, respectively. When the compressor is turned off, the main cause of the system irreversibilities can be attributed to the condenser. In this stage, the mass flows rates imposed by the compressor and through the capillary tube decay after a few minutes, with the compressor presenting only residual mass flow rates in both its inlet and outlet, and the mass flow rate through the capillary tube decaying to less than 1/5 of its value in the first minute of the off regime. As a consequence, the entropy generated in the capillary tube and the compressor also decay, since there is no power input or mass flow rate. Note that in the off period the condenser is exchanging heat with the environment and this produces the presented irreversibilities.

The analysis of Equation (53) suggests that the main component of the entropy generated in the compressor is the heat exchanged with the external environment. However, the compressor thermodynamic losses are due to the various internal irreversibilities taking place inside the compressor, also calculated by the specific entropy difference between compressor outlet and inlet. The sum of all these losses makes this component the one with the highest entropy generation of the refrigeration system. Some processes that generate entropy inside the compressor are: heat transfer inside the compression cylinder, back flow in the compressor valves and exit line, fluid friction in the compressor internal tubes and mufflers, friction between metals due to mechanical parts movement, thermal losses in the electrical motor, and others. Ref. [26] presents the calculation of the various commented exergy destruction rates for an open reciprocating refrigeration compressor.

**Figure 13:**
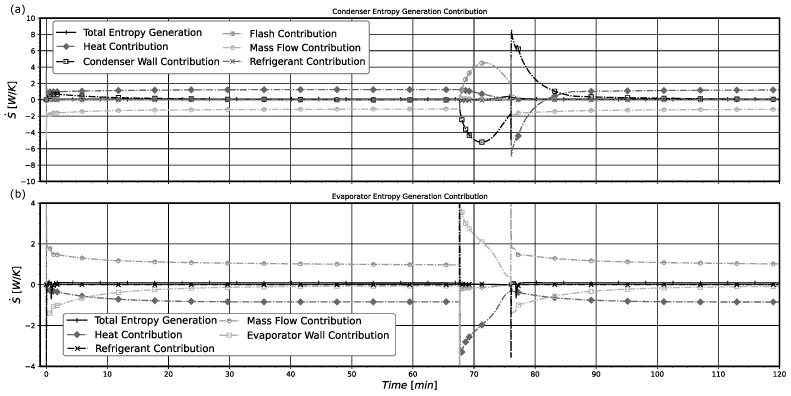
(**a**) Condenser entropy generation rate contributions. (**b**) Evaporator entropy generation rate contributions. Results obtained for model B.

In Figure 13 the rates of total entropy generation in the heat exchangers and the individual entropy variations that contribute to the total entropy generation rate obtained with model B are shown. Both heat exchangers show a similar behavior while the compressor is operating (turned on). In this state, the heat exchanged with the external environment (heat contribution) and the transport of entropy through the open frontiers (mass flow contribution) are the major contributions to the entropy generated within the systems. It is important to notice that in general these contributions play opposing roles, with one increasing the entropy generation (heat contribution for the condenser and mass flow contribution for the evaporator) and the other decreasing the entropy generation. When the system enters into an *off regime*, the entropy contributions made by the flashing evaporation (flash contribution) effect and the one due to the entropy variation of the solid component (condenser wall contribution) present similar behaviors with different signals. For the evaporator, the irreversibility can be attributed to both the heat flow into the system (heat contribution) and the change in the evaporator wall entropy (evaporator wall contribution). It should be noted that the condenser and evaporator total entropy generation rates are always positive. The terms denoted by the entropy temporal variations could be positive and negative. The results displayed in Figure 13 are very interesting and show in detail how the entropy is generated in the main system heat exchangers.

**Figure 14:**
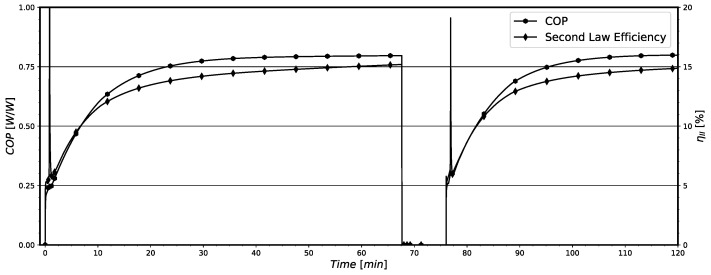
System instantaneous $\eta_{II}$ and COP for on-off operation for model B.

Last, the instantaneous values of $\eta_{II}$ and COP, estimated with model B for the system operation with time, are shown in Figure 14. The displayed results show that the system operates with instantaneous low efficiencies, being $\eta_{II}$ bellow 15% and the COP lower than 0.80. Furthermore, it is possible to notice that when the system starts to operate, i.e., the compressor is just turned on, the values for COP and $\eta_{II}$ are minimal, increasing with time. This effect was previously explained in Section 5.1 for the pull-down operation and can be associated with the irreversibility results shown in Figure 12. In the the initial moments, the system presents maximum irreversibilities due to higher temperature differences and mass flow rate variations. This behavior translates into a negative effect for the system efficiencies parameters, COP and $\eta_{II}$. However, in the on-off operation, the differences of temperatures and mass flow rates are lower than those for the pull-down operation. Then, both COP and $\eta_{II}$ show similar behavior with time, having similar augmentation rates of their values.

### 5.3. Effects of Thermal Load

In this section, how the presence of a thermal load inside the cabinet compartment affects the entropy generation during the system operation is evaluated. For this purpose, the Model B is used to simulate the presence of 10 kg of meat, at a constant rotation of 4500 rpm with 103 g of R290 for three environment temperatures, $T_{env}$ = 25 °C, 32 °C, and 43 °C, considering a total operation time of 2880 min or 2 days. In each simulation, the goods were initially at $T_{env}$ and were inserted into the compartment after a period of 1 day of operation. This was performed in order to let each simulation evolve to its steady state.

**Figure 15:**
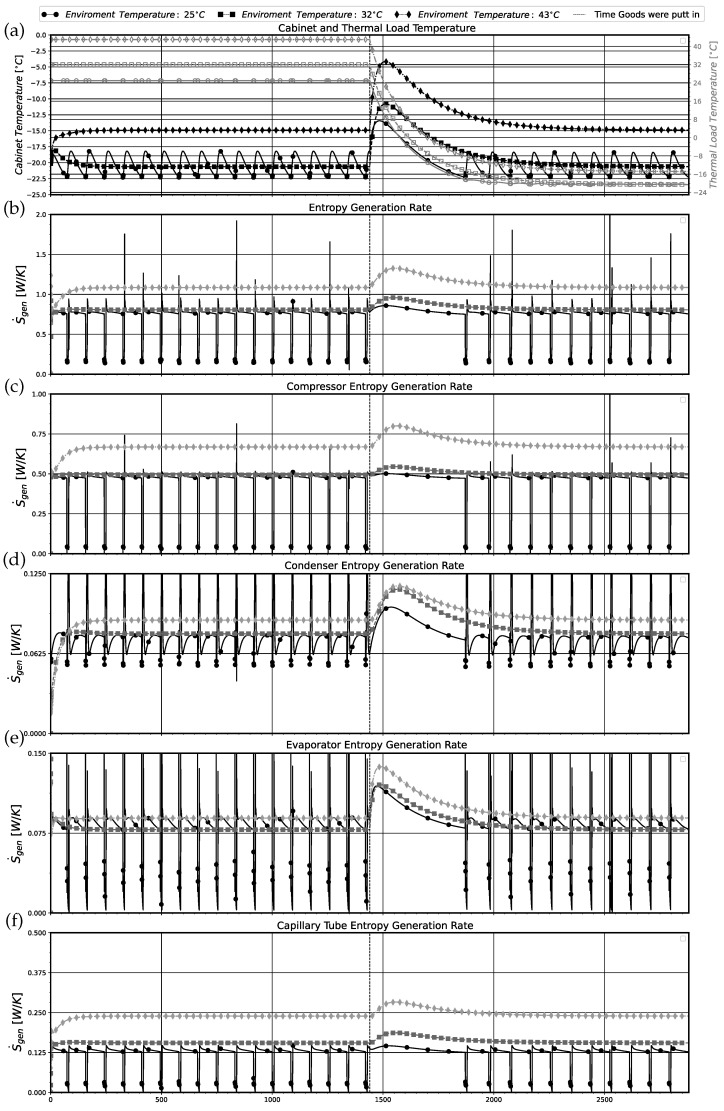
Simulated results with thermal load for the environmental temperatures of 25 °C (circle), 32 °C (square), and 43 °C (diamond). (**a**) Cabinet (solid symbols) and goods temperature (hollow symbol); (**b**) total entropy generation rate in the system; (**c**) entropy generation rate in the compressor; (**d**) entropy generation rate in the condenser; (**e**) entropy generation rate in the evaporator; (**f**) entropy generation rate in the capillary tube.

In Figure 15, the transient simulation results for the air inside the cabinet and goods’ temperatures (Figure 15a) and the entropy generation rates of the system (Figure 15b) and its components (Figure 15c–f) are shown. As can be seen in Figure 15, the simulations for $T_{env}=32$ °C and $T_{env}=43$ °C stayed with the compressor turned on the entire time, i.e., two days, mimicking the pull-down operation. For $T_{env}=32$ °C, the system successfully reached the allowed maximum cabinet temperature of −19.5 °C but failed to reach the minimum allowed cabinet temperature of −22.3 °C, staying in the desired temperature range. For $T_{env}=43$ °C, the system could not reach the allowed maximum cabinet temperature. This indicates that the studied dual-skin chest-freezer refrigeration system needs more refrigerant to properly operate at these two ambient temperatures. In these two cases, the entropy generation terms do not present variations with time. As expected, the entropy generation for the systems and it components were higher for $T_{env}=43$ °C in comparison with those calculated for $T_{env}=32$ °C.

In the case of $T_{env}=25$ °C, the system worked according to the on-off control logic, reaching the allowed maximum and minimum cabinet temperatures and staying in the desired temperature range. The entropy generation rates for this temperature were lower than those obtained for $T_{env}=43$ °C but were slightly higher than the obtained for $T_{env}=32$ °C. The physical reason for this behavior is explained later on. Similarly to the results presented in Figure 7 and Figure 12, the compressor was the main source of irreversibility in the system, followed by the capillary tube and the heat exchangers for all values of $T_{env}$. The insertion of the thermal load into the system results in a momentous increase in the entropy generation of all components. After this period, the goods were refrigerated and the system returned to the same operational regime corresponding to the empty system. This also concerned the entropy generated (see Figure 15).

The insertion of thermal loads in the system increased the cabinet internal air temperature, $T_{cab}$, resulting in an augmentation of the system temperature difference between $T_{cab}$ and $T_{we}$. This behavior provokes a greater entropy generation and, consequently, higher thermodynamic irreversibilities in the system. As values of $T_{cab}$ stabilize and return to the established temperature range (only for $T_{env}=25$ °C and 32 °C), the temperature difference is reduced, decreasing the entropy generation rates to the previous similar levels of the empty system. For $T_{env}=43$ °C, this also occurs, but in this case the values of $T_{cab}$ do not return to the desired temperature range.

In Table 15, time-averaged performance parameters for simulations with two environmental temperatures of $25^{{}^{\circ}}$ and $32^{{}^{\circ}}$ considering the entire time series are shown. The results present similar COP values, despite the fact that the simulation with $32^{{}^{\circ}}$ presents a higher energy consumption (and consumed compressor power). This behavior is justified by the continuous system operation for $T_{env}=32^{{}^{\circ}}$, without compressor cycling, allowing the system to operate steadily and to transfer more heat to the evaporator, ${\dot{Q}}_{e,avg}$. It should be noted that for $T_{env}=32^{{}^{\circ}}$, the system is not reaching the desired minimal value of $T_{cab}$.

Now, when analyzing the SLE it was noted that the simulation with higher $T_{env}$ produced a better efficiency $\eta_{II}$. This is associated with the effect of the on-off control that increased the system irreversibilities in the on periods for $T_{env}=25^{{}^{\circ}}$, leading to higher time average values of these irrevesibilities and consequently to a slightly smaller value of $\eta_{II}$. This behavior can be seen in Figure 15. Actually, for $T_{env}=25$ °C, every time that the compressor starts up again, the entropy generation rate in the system presents an overshoot for all system components. The cumulative effects of these overshoots increases the overall average irreversibilities in the system leading to a more inefficient operation and to lower $\eta_{II}$. Otherwise, for $T_{env}=32$ °C, the spike in the entropy generation rate only occurs in the very beginning of the load introduction into the system, followed by the stabilization and minimization of the irreversibilities in the system as it attain the stationary regime. This contradictory behavior for these two different values of $T_{env}$ indicates the necessity of taking care when SLE alone is used for optimizing the design of a refrigeration system. In this case, a system operating in a condition in which higher energy consumption is providing a better value of SLE, while it is not attaining the minimum $T_{cab}$ temperature and is not working under the desired control logic.

### 5.4. Model Limitations and Future Extensions

The present model is a lumped model with limitations represented by the adopted simplifying hypothesis, presented in Section 2. Future extensions of the present model include the development of a distributed model which will allow a better assessment of the thermodynamic and transport properties variation with temperature and pressure changes and also of the component surface temperatures. The simulation of connecting lines is also a point that will be addressed in future model developments. This will allow a better estimation of the refrigerant mass distribution in the refrigeration system components, considering also the simulation of transport delays. The construction of a distributed model with connecting lines should allow the calculation of pressure drops in the system components and connections. Last, the modeling and simulation of doors opening and air infiltration should be included in future versions of the model.

## 6. Conclusions

A refrigeration system of a dual-skin chest-freezer operating with R290 was simulated providing the transient characterization of the system behavior, in special, simulation results regarding thermodynamic irreversibilities obtained from an analysis of the second law of thermodynamics. System simulations in both pull-down and on-off operational regimes were performed for specific conditions and compared with measured experimental data. Very reasonable simulation results were achieved, with maximum RMS errors of 2% and 3% for the surface component temperatures in the pull-down and on-off operations. The proposed modified model (model B) presented slightly better results than the original model from [9] (model A), showing a maximum relative error of 2.05% for ${\dot{W}}_{avg}$. Transient system irreversibilities were calculated from simulations, showing that the compressor is the main source of the system irreversibilities, followed by the capillary tube and then by the heat exchangers. The total entropy generated in the heat exchangers was calculated, including the explicit determination of its main five particular sources, namely, condenser wall entropy variation, flash process entropy variation, entropy variation due to mass flow, entropy transfer due to heat transfer with the environment, and entropy variation of the refrigerant. This splitting calculation of total entropy generation in the heat exchangers allowed a more detailed analysis and a further understanding of how this components generates entropy. Actually, the entropy generation mechanisms are opposite in each heat exchanger. The calculated thermodynamic irreversibilities and entropy generation values with the realized numerical simulations were physically coherent with the expected observations from theory. The system irreversibilities are higher when the system is operating in a highly transient regime, i.e., just when the compressor is turned on or turned off. In this case, the system presents lower $\eta_{II}$ and COP. After these transients are over-passed and the system works in a more cyclic regime, the thermodynamic irreversibilities decrease and system $\eta_{II}$ and COP increase. This behavior indicates that turning the system on and off consecutively with a high frequency leads to lower COP and $\eta_{II}$ values, as the system does not attain a more continuous operation. Thus, correct selection of a compressor that would lead to a more efficient and continuous operation is very important. The study of thermal load influence on system performance showed that for the highest ambient temperatures, ($T_{env}=$ 43 °C), the system was unable to attain the desired low cabinet temperature of −19.5 °C. This malfunction indicated the necessity of increasing the refrigerant charge of the system, pointing out the utility of the employed simulation tool. Furthermore, the comparative results obtained with thermal load for $T_{env}=25$ °C and 32 °C, indicate that the system operating at the higher $T_{env}$ produced a better SLE value. However, this was an effect of the on-off control logic use that thermodynamically degraded the system operation under $T_{env}=25$ °C. This type of result should be properly analyzed and warns against the use of SLE criteria alone for the system optimization. The results for the entropy generation confirm that the compressor continued to be the higher source of irreversibilities of the system.

## Figures and Tables

**Table 1:** Equations for the compressor sub-model.

Mass flow rate	(1) m˙com=ηvolρ1V˙dispNRPM60
Electric power consumption	(2) $\dot{W}={\dot{m}}_{com}\frac{\left(h_{2s}-h_{1}\right)}{\eta_{gl}}$
Heat transfer rate through the housing	(3) ${\dot{Q}}_{com}=UA_{com}\left(T_{com}-T_{env}\right)$
Housing temperature	(4) $C_{com}\frac{dT_{com}}{dt}={\dot{W}}_{com}-{\dot{Q}}_{com}+{\dot{m}}_{suc}h_{1}-{\dot{m}}_{com}h_{2}$
Discharge temperature	(5) $T_{2}=\;\left(\frac{T_{1}+T_{com}}{2}\right){\left(\frac{P_{cond}}{P_{evap}}\right)}^{\frac{n_{p}-1}{n_{p}}}$

**Table 2:** Compressor Characterization.

Volumetric Efficiency (Reference Speed)	(8) $\eta_{Vol_{ref}}=a_{1}+a_{2}\cdot {\left[\frac{P_{cond}}{P_{evap}}\right]}^{1/k}$
Volumetric Efficiency	(9) $\eta_{Vol}=\left[a_{3}+a_{4}\cdot \left(\frac{N_{RPM}}{N_{RPM_{ref}}}\right)\;+\;a_{5}\cdot {\left(\frac{N_{RPM}}{N_{RPM_{ref}}}\right)}^{2}\right]\;\cdot \;\eta_{Vol_{ref}}$
Isentropic Efficiency (Reference Speed)	(10) ηglref=m˙com(h2s−h1)V˙Pevapb1·PcondPevapb2+k−1k+b3Pcond + W˙loss
Isentropic Efficiency	(11) $\eta_{gl}=\eta_{gl_{ref}}\cdot {\left[b_{4}+b_{5}\cdot \;\left(\frac{N_{RPM}}{N_{RPM_{ref}}}\right)\;+\;b_{6}\cdot {\left(\frac{N_{RPM}}{N_{RPM_{ref}}}\right)}^{2}\right]}^{-1}$
Overall Conductance	(12) $T_{2}=\frac{{\dot{W}}_{com}-{\dot{m}}_{com}\left(h_{2s}-h_{1}\right)}{UA_{com}}+T_{1}$

**Table 3:** Equations for the condenser sub-model.

External heat transfer rate	(13) ${\dot{Q}}_{C}={UA}_{C}\cdot \left[T_{env}-T_{wc}\right]$
Heat Exchanged with the refrigerant	(14) ${\dot{Q}}_{cond}={\bar{h}}_{cond}\cdot A_{ci}\cdot \left[T_{cond}-T_{wc}\right]$
Condenser Temperature Derivative	(15) $\frac{dT_{wc}}{dt}=\frac{{\dot{Q}}_{cond}+{\dot{Q}}_{C}-{\dot{Q}}_{flash}}{C_{C}}$
Mass Balance Equation	(16) $\frac{dM_{C}}{dt}={\dot{m}}_{com}-{\dot{m}}_{cap}\;$
Refrigerant Outlet Quality	(17) $x_{3}=\frac{M_{vlc}-M_{C}}{M_{vlc}-M_{vc}}$
Condenser Outlet Temperature	(18) $T_{3}=T_{env}+\left[T_{cond}-T_{env}\right]\cdot exp\left[\frac{-{UA}_{sc}}{{\dot{m}}_{cap\cdot c_{P_{l}}}}\right]$
Sub-Cooled External Heat Transfer Area	(19) $A_{sc}=A_{C}\cdot \left[\frac{M_{C}-M_{vlc}}{M_{lc}-M_{vlc}}\right]$
Sub-Cooled Conductance	(20) ${UA}_{sc}=\left(\frac{{UA}_{C}}{A_{C}}\right)\cdot A_{sc}$
Condensation Pressure	(21) dPconddt=dUCdt−f2·dMCdtVC·df1dP+Mc·df2dP
Refrigerant Total Internal Energy	(22) $\frac{dU_{C}}{dt}={\dot{m}}_{com}\cdot h_{2}-{\dot{m}}_{cap}\cdot h_{3}-{\dot{Q}}_{cond}$
Condensation Pressure when there is only gas	(23) dPconddt=Z·R·dUCdtcv·VC
Heat flux when flash occurs	(24) ${\dot{Q}}_{flash}={\bar{h}}_{cond}\cdot A_{flash}\cdot \left[T_{cond}-T_{wc}\right]$
Super-Heated Zone Heat Transfer Area	(25) $A_{sh}=A_{Ci}\cdot \left[\frac{M_{vlc}-M_{C}}{M_{vlc}-M_{vc}}\right]$
Flashing Zone Heat Transfer Area	(26) $A_{flash}=A_{Ci}\cdot \left[\frac{M_{c}-M_{vc}}{M_{vlc}-M_{vc}}\right]$

**Table 4:** Partial Derivatives.

$f_{1}$ Partial Derivative	(27) $$\frac{df_{1}}{dP}=3\beta_{1}P^{2}+2\beta_{2}P+\beta_{3}$$
$f_{2}$ Partial Derivative	(28) $$\frac{df_{2}}{dP}=\frac{\beta_{4}}{P}+\frac{\beta_{5}}{2}P^{-1/2}$$

**Table 5:** Partial Derivatives Coefficients.

Coefficients	
$\beta_{1}$	$1.23309\times 10^{-13}$
$\beta_{2}$	$-8.30757\times 10^{-7}$
$\beta_{3}$	6.89189
$\beta_{4}$	34721.6
$\beta_{5}$	116.016

**Table 6:** Equations for the evaporator sub-model.

External heat transfer rate	(30) ${\dot{Q}}_{E}={UA}_{E}\cdot \left[T_{cab}-T_{we}\right]$
Heat Exchanged with the refrigerant	(31) ${\dot{Q}}_{evap}={\bar{h}}_{evap}\cdot A_{ei}\cdot \left[T_{evap}-T_{we}\right]$
Evaporator Temperature Derivative	(32) $\frac{dT_{we}}{dt}=\frac{{\dot{Q}}_{E}+{\dot{Q}}_{evap}}{C_{E}}$
Mass Balance Equation	(33) $\frac{dM_{E}}{dt}={\dot{m}}_{cap}-{\dot{m}}_{com}$
Refrigerant Outlet Quality	(34) $x_{5}=\frac{M_{vle}-M_{E}}{M_{vle}-M_{ve}}$
Evaporator Outlet Temperature	(35) $T_{5}=T_{cab}+\left[T_{evap}-T_{cab}\right]\cdot exp\left[\frac{-{UA}_{sh}}{{\dot{m}}_{com}c_{p,v}}\right]$
Super-heated conductance	(36) ${UA}_{sh}=\left(\frac{{UA}_{E}}{A_{E}}\right)\cdot A_{sh,e}$
Super-heated External Heat Transfer Area	(37) $A_{sh,e}=A_{E}\cdot \left[\frac{M_{E}-M_{vle}}{M_{ve}-M_{vle}}\right]$
Evaporation Pressure	(38) dPevapdt=dUEdt−f2·dMEdtVE·df1dP+ME·df2dP
Refrigerant Total Internal Energy	(39) $\frac{dU_{E}}{dt}={\dot{m}}_{cap}\cdot h_{4}-{\dot{m}}_{com}\cdot h_{5}-{\dot{Q}}_{evap}$

**Table 7:** Equations for the cabinet sub-model.

Heat transfer rate through the walls	(40) ${\dot{Q}}_{cab}={UA}_{cab}\cdot \left(T_{env}-T_{cab}\right)$
Temperature inside freezer	(41) $\frac{dT_{cab}}{dt}=\frac{{\dot{Q}}_{cab}+{\dot{Q}}_{g}-{\dot{Q}}_{E}}{C_{cab}}$
Heat transfer rate to the goods	(42) ${\dot{Q}}_{g}={UA}_{g}\cdot \left(T_{g}-T_{cab}\right)$
Goods Temperature	(43) $\frac{dT_{g}}{dt}=\frac{-{\dot{Q}}_{g}}{C_{g}}$

**Table 8:** Entropy generation and exergy destruction equations for the compressor.

Compressor Exergy Destruction Rate	(52) ${\dot{X}}_{des,com}=\dot{W}+{\dot{m}}_{suc}\cdot \;\left[h_{1}-T_{env}s_{1}\right]\;-\;{\dot{m}}_{com}\cdot \;\left[h_{2}-T_{env}s_{2}\right]\;-\;\frac{d}{dt}X_{com,hous}$
Compressor Entropy Generation Rate	(53) ${\dot{S}}_{gen,com}={\dot{m}}_{com}s_{2}-{\dot{m}}_{suc}s_{1}+\frac{{\dot{Q}}_{com}}{T_{env}}+\frac{d}{dt}S_{com,hous}$
Compressor Housing Exergy Variation with time	(54) $\frac{d}{dt}X_{com,hous}=\frac{d}{dt}U_{com,hous}-T_{env}\cdot \frac{d}{dt}S_{com,hous}$
Compressor Housing Entropy Variation with time	(55) $\frac{d}{dt}S_{com,hous}=\frac{1}{T_{com}}\cdot \frac{d}{dt}U_{com,hous}$

**Table 9:** Exergy destruction and entropy generation for the capillary tube.

Capillary Exergy Destruction Rate	(56) ${\dot{X}}_{des,cap}={\dot{m}}_{cap}\cdot T_{env}\cdot \;\left[s_{4}-s_{3}\right]$
Capillary Entropy Generation Rate	(57) ${\dot{S}}_{gen,cap}={\dot{m}}_{cap}\cdot \left[s_{4}-s_{3}\right]$

**Table 10:** Exergy destruction and entropy generation for the Heat Exchangers.

Total Exergy Destruction Rate	(58) ${\dot{X}}_{des}={\dot{m}}_{in}\cdot \;\left[h_{in}-T_{env}s_{in}\right]\;-\;{\dot{m}}_{out}\cdot \;\left[h_{out}-T_{env}s_{out}\right]\;-\;\frac{d}{dt}X_{VC}$
Total Entropy Generation Rate	(59) ${\dot{S}}_{gen}={\dot{m}}_{out}\cdot s_{out}-{\dot{m}}_{in}\cdot s_{in}-\frac{{\dot{Q}}_{wall}}{T_{env}}+\frac{d}{dt}S_{VC}$
Total Exergy Change with time	(60) $\frac{d}{dt}X_{VC}=\frac{d}{dt}U_{ref}+\frac{d}{dt}U_{wall}-T_{env}\cdot \left[\frac{d}{dt}S_{ref}+\frac{d}{dt}S_{wall}\right]$
Wall Entropy Change with time	(61) $\frac{d}{dt}S_{wall}=\frac{1}{T_{wall}}\cdot \frac{d}{dt}U_{wall}$

**Table 11:** Thermal conductances and capacities of systems components.

	Compressor	Condenser	Evaporator	Cabinet
Capacitance [kJ K${}^{-1}$]	4.48±0.33	48.32±3.91	18.74±1.49	23.33±1.66
Thermal Conductance [W K${}^{-1}$]	3.44	26.85±2.17	15.62±1.24	4.05±0.29

**Table 12:** Pull-Down steady-state relative errors and time series RMS errors regarding the experimental data.

	Model A		Model B	
	Steady-State Error [%]	Time Series Error [%]	Steady-State Error [%]	Time Series Error [%]
$T_{com}$	0.88	1.15	0.17	1.02
$T_{wc}$	0.15	0.79	0.22	0.81
$T_{we}$	0.31	1.01	0.07	0.94
$T_{cab}$	0.27	1.93	0.02	1.89
$P_{cond}$	1.16	6.80	1.66	6.64
$P_{evap}$	3.93	21.46	0.82	21.34

**Table 13:** Average performance parameters obtained by simulations and experimental tests in the on-off operation.

	COP [W/W]	Consumption [kWh month^−1^]	${\dot{Q}}_{e,\mathrm{avg}}$ [W]	${\dot{W}}_{\mathrm{com},\mathrm{avg}}$ [W]
Experimental	0.65±0.05	222.50±3.34	201.01±14.34	309.03±4.64
Model A	0.63	216.22	189.41	300.31
Model B	0.63	217.92	189.33	302.67

**Table 14:** On-off time series RMS errors.

	$T_{\mathrm{com}}$ [%]	$T_{\mathrm{wc}}$ [%]	$T_{\mathrm{we}}$ [%]	$T_{\mathrm{cab}}$ [%]
Model A	1.07	2.01	1.37	0.42
Model B	1.05	1.96	1.30	0.41

**Table 15:** Average performance parameters obtained by simulations with model B and thermal load.

	COP [W/W]	$\eta_{\mathrm{II}}$ [%]	Consumption [kWh month^−1^]	${\dot{Q}}_{e,\mathrm{avg}}$ [W]	${\dot{W}}_{\mathrm{com},\mathrm{avg}}$ [W]
$T_{env}=25$ °C	0.63	13.65	222.87	195.65	309.54
$T_{env}=32$ °C	0.63	15.01	249.07	218.95	345.93

## Data Availability

Not applicable.

## Abbreviations

The following abbreviations are used in this manuscript:

*A*	Area, [m${}^{2}$]
$c_{P}$	specific heat at constant pressure, [J kg${}^{-1}$ K${}^{-1}$]
*C*	thermal capacity, [kJ K${}^{-1}$]
COP	coefficient of performance
VCS	vapor compression cycles
CV	control volume
SLE	second law efficiency
RMS	root mean square
*h*	specific enthalpy, [J kg${}^{-1}$]
*s*	specific entropy, [J kg${}^{-1}$ K${}^{-1}$]
*M*	mass, [kg]
$\dot{m}$	mass flow rate, [kg s${}^{-1}$]
$N_{RPM}$	rotation, [rpm]
$n_{p}$	polytropic exponent
*t*	time, [s]
*T*	temperature, [°C]
*P*	pressure, [kPa]
$\dot{Q}$	heat transfer rate, [W]
*R*	gas constant, [J kg${}^{-1}$ K${}^{-1}$]
$\dot{W}$	electric power, [W]
*U*	total internal energy, [J]
*u*	specific internal energy, [J kg${}^{-1}$]
UA	thermal conductance, [W K${}^{-1}$]
$\bar{h}$	average heat transfer coefficient, [W m${}^{-2}$ K${}^{-1}$]
*v*	specific volume, [m${}^{3}$ kg${}^{-1}$]
V	volume, [m${}^{3}$]
*x*	quality
${\dot{S}}_{gen}$	entropy generation rate, [W K${}^{-1}]$
${\dot{X}}_{des}$	exergy destruction rate, [W]
${\dot{W}}_{rev}$	reversible work per time, [W]
*Z*	compressibility factor
$f_{1}$	property relation defined in the text, [J m${}^{-3}$]
$f_{2}$	property relation defined in the text, [J kg${}^{-1}$]
$\beta_{i}$	fitted coefficient for $f_{i}$ calculation
α	void fraction
Δ	variation
η	efficiency
ρ	density, [kg m${}^{-3}$]
β	(1, 2, 3, 4, and 5) fitting coefficients presented in Table 4 and Table 5,
env	environment
*C*	condenser
wc	condenser wall, condenser surface
cab	cabinet, compartment
cap	capillary tube
com	compressor
cond	condensation
*d*	discharge
disp	displacement
*E*	evaporator
we	evaporator wall, evaporator surface
evap	evaporation
*g*	goods
gl	global
in	inlet
out	outlet
*l*	liquid
*v*	vapor
sat	saturation
sc	sub-cooled
sh	super-heating
vol	volumetric
*i*	state point (1, 2, 3 …)
II	second law
suc	compressor suction side
shell	compressor suction chamber
ref	reference
hous	compressor housing
2s	isentropic state 2
${}^{{}^{\circ}}$	values at past time iteration
